# Rationales Design von Phe‐BODIPY‐Aminosäuren als fluorogene Bausteine für den peptidbasierten Nachweis von *Candida*‐Infektionen im Harntrakt

**DOI:** 10.1002/ange.202117218

**Published:** 2022-02-26

**Authors:** Lorena Mendive‐Tapia, David Mendive‐Tapia, Can Zhao, Doireann Gordon, Sam Benson, Michael J. Bromley, Wei Wang, Jun Wu, Adelina Kopp, Lutz Ackermann, Marc Vendrell

**Affiliations:** ^1^ Zentrum für Entzündungsforschung Die Universität von Edinburgh EH16 4TJ Edinburgh Großbritannien; ^2^ Abteilung Theoretische Chemie Physikalisch-Chemisches Institut Universität Heidelberg 69120 Heidelberg Deutschland; ^3^ Manchester Fungal Infection Group Abteilung für Evolution Infektion und Genomik M139NT Manchester Großbritannien; ^4^ Institut für Organische und Biomolekulare Chemie Georg-August-Universität 37077 Göttingen Deutschland

**Keywords:** Aminosäure, BODIPY, Candida, Fluoreszenz, Sonde, TD-DFT, Angeregten Zustand

## Abstract

Pilzinfektionen, die durch Candida‐Arten verursacht werden, gehören zu den häufigsten Infektionen bei Krankenhauspatienten. Die derzeitigen Methoden zum Nachweis von Candida‐Pilzzellen in klinischen Proben beruhen jedoch auf zeitaufwändigen Analysen, die eine schnelle und zuverlässige Diagnose erschweren. In diesem Beitrag beschreiben wir die rationale Entwicklung neuer Phe‐BODIPY‐Aminosäuren als kleine fluorogene Bausteine und ihre Anwendung zur Erzeugung fluoreszierender antimikrobieller Peptide für die schnelle Markierung von Candida‐Zellen im Urin. Mit Hilfe von computergestützten Berechnungen haben wir das fluorogene Verhalten von BODIPY‐substituierten aromatischen Aminosäuren analysiert und Bioaktivitäts‐ und konfokale Mikroskopieexperimente bei verschiedenen Stämmen durchgeführt, um den Nutzen und die Vielseitigkeit von Peptiden mit Phe‐BODIPYs zu bestätigen. Schließlich haben wir einen einfachen und sensitiven fluoreszensbasierten Test zum Nachweis von Candida albicans in menschlichen Urinproben entwickelt.

## Graphical abstract

Wir beschreiben das rationale Design und die Synthese der ersten fluorogenen BODIPY‐Aminosäuren auf Phe‐Basis und ihre Anwendung zur Herstellung von peptidbasierten Wirkstoffen für die schnelle und waschfreie Markierung von Pilzzellen. Dies wurde genutzt, um einen sensitiven und kostengünstigen Fluoreszenztest für den Nachweis von *Candida albicans* in menschlichem Urin unter Verwendung einfacher Tisch‐Spektralphotometern zu entwickeln.

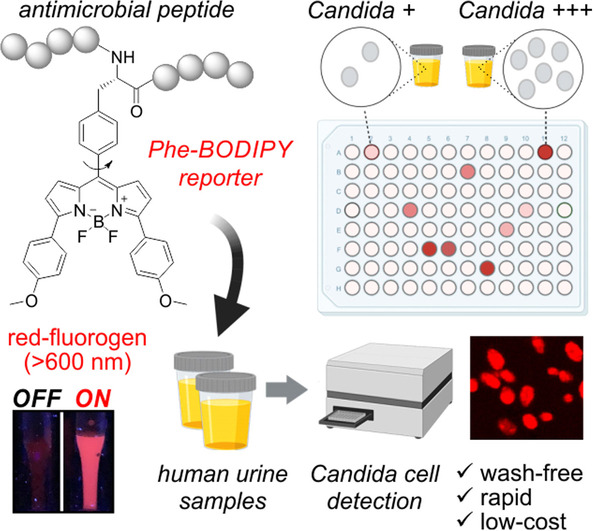

## Einleitung

Die Diagnose invasiver Pilzinfektionen ist nach wie vor ein wichtiges Thema im öffentlichen Gesundheitswesen. In den letzten Jahren hat das Auftreten arzneimittelresistenter Pilze die Häufigkeit von im Krankenhaus erworbenen Pilzinfektionen deutlich erhöht.^[1]^ Infektionen, die durch Pilzerreger der Spezies *Candida* verursacht werden, gehören zu den am häufigsten diagnostizierten Infektionen bei Krankenhauspatienten^[2]^ und führen zu oberflächlichen (z. B. Soor) oder disseminierten systemischen Infektionen (z. B. Septikämie).^[3]^ Herkömmliche Assays zum Nachweis *Candida*‐Zellen beruhen auf Histopathologie, Polymerase‐Kettenreaktionen und Zellkulturen, die aufgrund zeitaufwändiger und mehrstufiger Protokolle mehrere Tage in Anspruch nehmen können.^[4]^ Infolgedessen verzögert sich die Verschreibung geeigneter Behandlungen häufig und kann zu unerwünschten Nebenwirkungen führen, einschließlich Multiresistenzen aufgrund eines übermäßigen Einsatzes empirischer Antibiotika.

Diese Einschränkungen haben dazu geführt, dass einfachere und schnellere Ansätze für den Nachweis von Krankheitserregern in klinischen Proben entwickelt wurden.^[5]^ In diesem Zusammenhang sind Urinproben aufgrund ihrer Zugänglichkeit und ihrer direkten Relevanz für Harnwegsinfektionen attraktiven klinischen Proben. Das Vorhandensein von *Candida*‐Zellen im Urin (d.h. Candiduria) ist eine häufige nosokomiale Infektion, die 5–10 % aller positiven Urinkulturen in Krankenhäusern und tertiären Pflegeeinrichtungen ausmacht.^[6]^ Obwohl *Candida* der am häufigsten vorkommende Pilz bei Patienten mit UT‐Infektionen ist,[^2b^, ^7^] gibt es keine klinisch zugelassenen Tests für den direkten Nachweis von Candiduria.^[8]^

Die optische Bildgebung ist ein leistungsfähiges Instrument zur Untersuchung wesentlicher biologischer Prozesse in Zellen.^[9]^ Daher haben die jüngsten Fortschritte in der Urinanalyse zur Entwicklung von Assays geführt, die Fluoreszenzanzeigen für den Nachweis von Bakterien und anderen Krankheits‐Biomarkern verwenden.^[10]^ Die Assays verwenden fluoreszenzmarkierte molekulare Reporter, deren Emission bei Erkennung eines spezifischen Analyten (z. B. Lipide, Enzyme) verstärkt wird.^[11]^ Das Aufkommen fluoreszierender Aminosäuren (FIAAs) für die Konstruktion peptidbasierter Bildgebungsreagenzien hat das Moleküldesign, die auf pathogene Zellen abzielen, beschleunigt.^[12]^ Unsere Gruppe hat zur Optimierung der Übergangsmetallkatalysierten Synthesemethoden für FIAAs^[13]^ sowie zu deren Anwendung in der biologischen Bildgebung beigetragen.^[14]^ Insbesondere die Entwicklung von FIAAs mit fluorogenem Charakter hat sich als erfolgreich erwiesen, um bei Bioimaging‐Experimenten ein hohes Signal‐Rausch‐Verhältnis zu erzielen.^[15]^ In dieser Arbeit haben wir neue fluorogene Aminosäuren auf der Grundlage des Phenylalanin (Phe)‐Kerns rational entworfen und sie zur Synthese von peptidbasierten Wirkstoffen für die schnelle und direkte Identifizierung von *Candida*‐Pilzzellen in menschlichen Urinproben unter Verwendung von Tisch‐Spektrophotometern genutzt.

## Ergebnisse und Diskussion

### Synthese und Charakterisierung von BODIPY FIAAs

**Figure 1:**
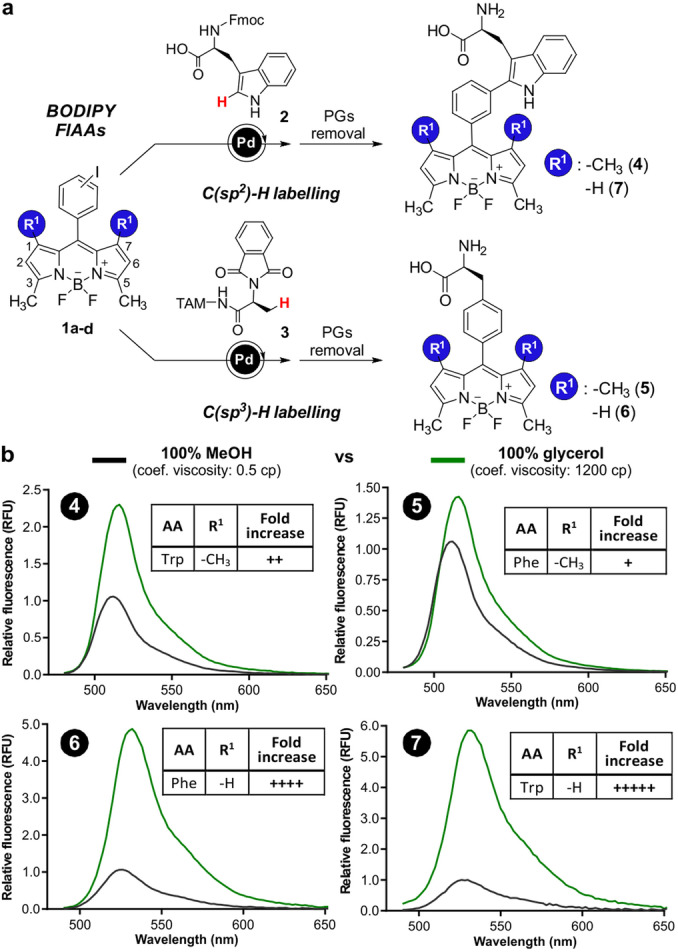
a) Synthetisches Schema für die Präparation von Trp und Phe‐BODIPY‐Aminosäuren **4**–**7**. b) Fluorogenes Verhalten der Aminosäuren **4**–**7** (20 μM) in MeOH (schwarz) und Glycerin (grün). *λ*_exc_: 450 nm (für **4** und **5**) und 460 nm (für **6** und **7**) (*n*=3).

Das Gerüst aus Borondipyrromethan (BODIPY) ist aufgrund seiner hervorragenden photophysikalischen Eigenschaften ein attraktives Fluorophor für die Entwicklung von Bildgebungssonden.^[16]^ Zu den aktuellen Ansätzen für die Synthese von BODIPY‐Peptid‐Konjugaten gehören die klassischen CuAAC‐^[17]^ und SPAAC‐^[18]^ Methoden unter Verwendung vorfunktionalisierten Zwischenprodukte, Festphasenansätze für die in situ Dipyrrinkonstruktion von *N*‐terminalen/Lysinen^[19]^ und die Verwendung von BODIPY‐tragenden FIAAs,^[12a]^ wobei letztere den *site*‐selektiven Einbau des Fluorophors ermöglichen, ohne die nativen biomolekularen Eigenschaften der Peptide zu beeinträchtigen. Unter den verschiedenen BODIPY‐Strukturen, die für die Konstruktion von FIAAs berichtet wurden, sticht das fluorogene Trp‐BODIPY (**4**, Abbildung 1a) wegen seiner Nützlichkeit für die waschfreien Bildgebung biologischer Systeme hervor.^[20]^ Allerdings emittiert Trp‐BODIPY im grünen Bereich des sichtbaren Spektrums (≈520 nm), was intensitätsbasierte Messungen in klinischen Proben mit einer gewissen Autofluoreszenz (z. B. Urin) einschränken kann. Außerdem kann Trp aufgrund der geringen Anzahl von Trp‐Resten in kurzen bioaktiven Peptiden bei Markierungsexperimenten nicht direkt ersetzt werden. Andererseits ist Phe ein häufig vorkommender Rest in Peptiden, darunter auch in antimikrobiellen Peptiden, wo es eine Schlüsselrolle bei der Interaktion mit der Zellmembran und der anschließenden Zerstörung spielt.^[21]^ Mit diesem Hintergrund beschlossen wir, die Entwicklung kleinerer BODIPY‐FIAAs zu erforschen, die auf dem häufiger vorkommenden aromatischen Phe basieren und sich leicht in rot emittierende Reporter (>600 nm) umwandeln lassen, wo die Hintergrundfluoreszenz von Urin gering ist.

Zunächst haben wir die Phe‐BODIPY‐Aminosäure **5** (Abbildung 1a) hergestellt, die der Struktur von Trp‐BODIPY **4** ähnelt. Bei dieser Synthese verwendeten wir eine Pd‐katalysierte C(sp^3^)‐H‐Arylierung,^[22]^ um ein TAM‐geschütztes Alanin‐Surrogat und ein tetramethyliodiertes BODIPY‐Gerüst zu verbinden. Nach der Isolierung der freien Aminosäure **5**, untersuchten wir ihre Reaktion in Umgebungen mit unterschiedlicher Viskosität (d.h. Glycerin gegenüber Methanol mit 1200 bzw. 0.5 cp) und verglichen sie mit dem Trp‐BODIPY‐Gegenstück **4**, das mit zuvor beschriebenen Methoden gewonnen wurde (13d, 20b). Interessanterweise führte der einfache Ersatz von Trp durch Phe zu einer geringeren Fluorogenität, was den Nutzen von Verbindung **5** für die waschfreie Abbildung von Zellen einschränkte (Abbildung 1b). Um die Umweltempfindlichkeit von Phe‐BODIPY‐Aminosäuren zu verbessern, gingen wir davon aus, dass die Entfernung der Methylgruppen in den Positionen 1 und 7 der BODIPY‐Struktur die Fluorogenität der Verbindung verbessern könnte, ohne den Aminosäurekern oder den synthetischen Ansatzu zu verändern. Daher verwendeten wir die gleiche Synthesestrategie, um die Phe‐BODIPY‐Aminosäure **6** (Abbildung 1a) aus dem dimethylierten BODIPY‐Vorläufer zu gewinnen. Parallel dazu haben wir auch das dimethylierte Trp‐BODIPY (**7**, Abbildung 1a) synthetisiert, um die Auswirkungen der Substitution in Trp‐basierten FIAAs zu analysieren. Bemerkenswert ist, dass das Phe‐BODIPY **6** einen starken fluorogenen Charakter aufwies, mit einer Fluoreszensantwort, die der von Trp‐BODIPY ähnlich war, während die Gesamtgröße der FIAAs reduziert wurde.

### Spektroskopische und computergestützte Studien

**Figure 2:**
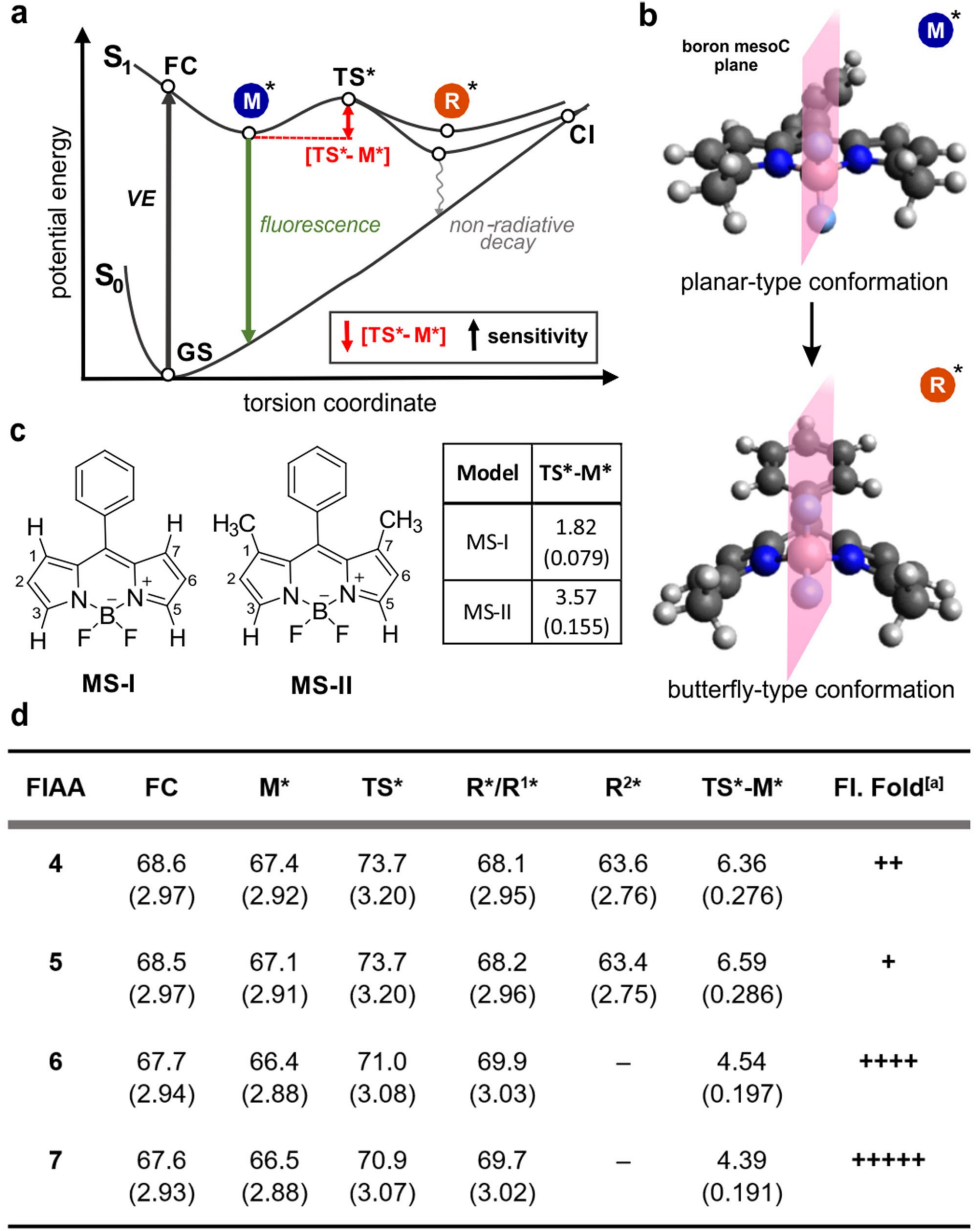
a) Vorgeschlagener Mechanismus für den S_1_ angeregten Zustand Reaktivität der BODIPY FlAAs. b) Repräsentatives Beispiel der optimierten Geometrien der angeregten Zustände für die M*‐ und R*‐Minimum‐Konformationen der BODIPY‐FIAAs.. c) Chemische Struktur der BODIPY Modellsysteme MS‐I und MS‐II und die dazu gehörigen TS*‐M* Energiebarrieren. d) FC, M*, TS* und R* Werte für die Aminosäuren **4**‐**7**. Für die Substrate **6** und **7**, ist die R* Konformation ein Minimum, während es sich bei den Substraten **4** und **5** um einen Übergangszustand (Cs‐Symmetrie) handelt, dessen imaginäre Normalschwingung die Symmetrie in zwei Konformationen mit niedriger Energie (R^1^* und R^2^*) bricht.^[a]^ Experimenteller Anstieg der Faltung, beobachtet in Abbildung 1b. d) Energiewerte in kcal mol^−1^ oder in eV (Klammern).

Um den Mechanismus hinter dem Verhalten der verschiedenen BODIPY‐basierten Aminosäuren zu verstehen, untersuchten wir die Beziehung zwischen den Verbindungen **4**‐**7** und ihrer Fluorogenität mit Hilfe von computergestüzten Berechnungen. Auf der Grundlage früherer Studien mit anderen BODIPY‐Farbstoffen,^[23]^ untersuchten wir die potenziellen Energieflächen (PESs) des Grundzustandes (S_0_) und des ersten angeregten Singulett‐Zustands (S_1_) zusammen mit den Torsionskoordinaten der Phenylgruppe. Wie in Abbildung 2 dargestellt, entspannt sich die Grundzustandsgeometrie (VE) von S_0_ zu S_1_ in der Frank‐Condon‐Region (FC), in der Konformation mit minimaler Energie (M*). Der Übergangszustand (TS*) beschreibt die Rotation der Phenylgruppe und steuert die Zugänglichkeit zu einer zweiten Reihe von Konformationen (R*/R^1^‐R^2^*) und einer konischen Kreuzung (CI) nahe S_1_/S_0._ Infolgedessen kann die Relaxation des angeregten Fluorophors zwei möglichen Wegen folgen: 1) Durch den CI und mit interner Energieumwandlung, bekannt als nicht‐strahlender Zerfall,^[24]^ oder 2) durch strahlenden Zerfall als Fluoreszenz, wenn die TS‐Energiebarriere groß genug ist, um den Zugang zum CI‐Übergang zu verhindern.^[25]^ Nach der Übergangszustandstheorie von Grote‐Hynes,^[26]^ steht die Umweltempfindlichkeit in direktem Zusammenhang mit dem Überschreiten einer niederfrequenten Barriere. Insbesondere werden PES mit niedrigen Barrieren mit Umgebungen in Verbindung gebracht, in denen die Reibungskräfte des Lösungsmittels dominieren.^[27]^ BODIPY‐Fluorophore, bei denen der Zugang zu einem CI und der anschließende nicht‐radiative Zerfall durch den Übergangszustand auf dem ersten angeregten Zustand kontrolliert wird, würde die entsprechende Barriere ihren fluorogenen Charakter bestimmen.^[28]^ Daher stellten wir die Hypothese auf, dass die Fluorogenität von BODIPY FIAAs mit ihren relativen angeregten Energiebarrieren zusammenhängen könnte, d.h. mit der S_1_‐Energiedifferenz zwischen TS* und der M*‐Konformation (d.h., TS*‐M*) (Abbildung 2a). Um dies zu bewerten, optimierten wir die relevanten stationären Punkte auf den PESs (z. B., FC, M*, TS* und R*) in voller Dimensionalität, anstatt nur entlang der Torsionskoordinate zu scannen, unter Verwendung der zeitabhängigen Dichtefunktionaltheorie (TD‐DFT) mit dem hybriden Austausch‐Korrelations‐Funktional M06‐2X.

Zunächst analysierten wir die TS*‐Barrieren im angeregten Zustand für eine Reihe von Modellsystemen analog zu den Aminosäuren **4**‐**7** (Abbildung 2d). Wir beobachteten eine Verringerung der Energiebarriere des dimethylierten Phe‐BODIPY **6** im Vergleich zum tetramethylierten Phe‐BODIPY **5** (4.54 kcal mol^−1^ beziehungsweise 6.59 kcal mol^−1^), was die erhöhte Umgebungsempfindlichkeit des ersteren erklärt. Ein ähnlicher Trend wurde für Trp‐BODIPY‐Verbindungen **4** und **7 (**6.36 kcal mol^−1^ vs. 4.39 kcal mol^−1^) beobachtet, wobei das dimethylierte Analogon niedrigere Energiebarrieren und eine stärkere Fluorogenität aufweist. Die Trp‐BODIPYs **4** und **7** wiederum zeigten niedrigere Energiebarrieren als die Phe‐BODIPYs **5** und **6**, was möglicherweise auf einen positiven Ladungstransfereffekt der Indolgruppe zurückzuführen ist. Um weitere Erkenntnisse über die Anordnung der TS‐Barrieren und die Auswirkungen von Methylgruppen zu gewinnen, analysierten wir auch zwei zusätzliche Modellsysteme, bei denen eine Phenyl‐BODIPY‐Struktur entweder Wasserstoff (MS‐I) oder Methylgruppen (MS‐II) in den Positionen 1 und 7 (Abbildung 2c) trägt. Das unsubstituierte MS‐I zeigte einen kleinen Energiebarrierenwert (1.82 kcal mol^−1^) und eine imaginäre Frequenz im Übergangszustand (64.69 i cm^−1^), welche auf eine flache Krümmung mit starker Umweltempfindlichkeit hindeuten. Andererseits wies MS‐II eine erhöhte Energiebarriere (3.57 kcal mol^−1^) auf, was wahrscheinlich auf die sterische Hinderung der Methylgruppen zurückzuführen ist (Abbildung 2c und ergänzende Tabelle S1). Die Methylierung an den Positionen 3 und 5 der Phe‐BODIPY‐Aminosäuren **6** führte ebenfalls zu einem größeren Anstieg der Barriere (4.54 kcal mol^−1^). Schließlich führte die tetramethylierte Phe‐BODIPY‐Aminosäure **5** zu einem fast additiven Effekt, der in der größten Barriere (6.59 kcal mol^−1^) aller Verbindungen gipfelte. Diese Barrieren wurden auch unter Verwendung des *polarizable continuum model* (PCM) Solvatationsschemas für Lösungsmittel mit unterschiedlichen Dielektrizitätskonstanten mit ähnlichen Ergebnissen berechnet (ergänzende Tabellen S2, S3). Die Optimierung der Geometrien bestätigte auch, dass die Trp‐BODIPY‐ und Phe‐BODIPY‐Aminosäuren bei der Anregung von einer planaren Konformation zu einer schmetterlingsartigen Konformation übergehen, mit einer erheblichen Phenylrotation und einer Biegung des BODIPY‐Kerns auf der vertikalen Bor‐MesoC‐Ebene (Abbildung 2b). Insgesamt bestätigen diese Beobachtungen, dass das Fehlen der Methylgruppen in Positionen 1 und 7 von Phe‐BODIPY‐FIAAs den Übergang zum Schmetterlingsminimum R* erleichtert, was aufgrund der geringeren TS*‐Barriere und der flachen Krümmung des PES zu einer erhöhten Umweltempflindlichkeit führt.

**Figure 3:**
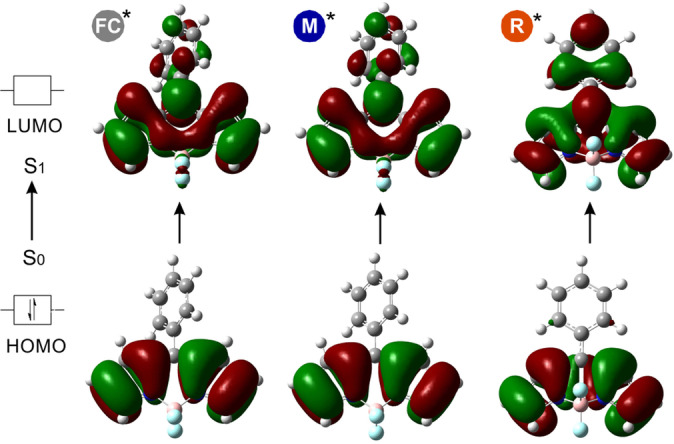
Dominante NTOs beim S_0_→S_1_ Übergang von MS‐I in den Geometrien von FC, M* und R*. Die entsprechenden NTO‐Eigenwerte sind ≥0.99, was bedeutet, dass die Anregung durch ein dominantes Anregungspaar gut beschrieben werden kann.

Als Nächstes untersuchten wir, ob ein intramolekularer Ladungstransfer (ICT) mit den optischen Eigenschaften der BODIPY FIAAs in Verbindung gebracht werden kann. In Anbetracht der Tatsache, dass natürliche Übergangsorbitale (NTO) eine gute qualitative Beschreibung der Veränderung des,^[29]^ verglichen wir diese für die FC‐, M*‐ und R*‐Geometrien von MS‐I bei Anregung von S_0_ zu S_1_ (Abbildung 3). Die jeweiligen HOMOs und LUMOs zeigten das Vorhandensein eines π‐π*‐Übergangs mit ICT vom BODIPY‐Kern zur Benzolgruppe. Dieser ist bei der R*‐Geometrie aufgrund der stärkeren Konjugation zwischen Donor‐ und Akzeptoranteilen, die in derselben Ebene ausgerichtet sind, stäker ausgeprägt. Diese Analyse zeigt, dass der Abschnitt des PES auf der M*‐Seite des TS* nur einen geringen Ladungstransfer im Vergleich zum Grundzustand aufweist, während dieser im Abschnitt auf der R*‐Seite erheblich ist. Daher sollten elektronenschiebende oder elektronenziehende Substituenten eine größere Wirkung auf den letztgenannten Bereich haben, indem sie die PES in Bezug auf den FC‐Bereich stabilisieren bzw. destabilisieren. In Übereinstimmung mit ähnlichen Analysen, die auf der Ebene ADC‐Theorie durchgeführt wurden,^[23]^ erklären diese Ergebnisse, dass einige Substituenten den TS* stabilisieren, da sich der Phenyl‐ und BODIPY‐Gruppen in diesem stationären Punkt teilweise überlappen.

Insgesamt deuten unsere Berechnungsstudien darauf hin, dass der fluorogene Charakter der Phe‐BODIPY‐Aminosäuren aus Substitutionsmustern resultiert, die durch die Analyse von Übergangszustandsbarrieren und NTOs auf der TD‐DFT‐Theorieebene mechanistisch rationalisiert werden können.

### Synthese und Bewertung von fluorogenen Peptiden für Candida‐Spezien

**Figure 4:**
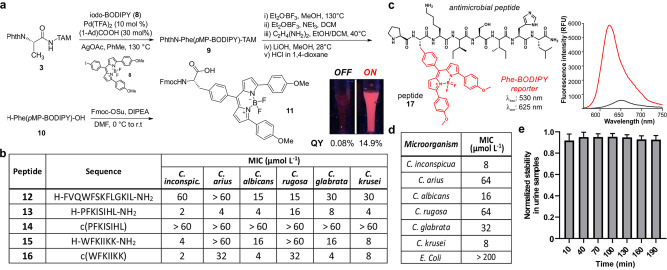
a) Synthetisches Verfahren zur Herstellung von Fmoc‐Phe(*p*MP‐BODIPY)‐OH (**11**). Inset: Bilder von Lösungen der Probe **17** (30 μM) unter Anregung mit einer 365 nm UV‐Lampe in PBS (links) und in Liposomen (rechts). b) Minimale Hemmkonzentration (MIC) der Peptide **12**–**16** gegen verschiedene *Candida‐*Stämme (5×10^5^ Zellen mL^−1^) in 20 % Vogel's‐Medium nach 48 h Inkubation bei 37 °C. Die MIC wurde mittels Hellfeldmikroskopie aus drei unabhängigen Experimenten bestimmt. c) Chemische Struktur von Peptid **17** und fluorogene Reaktion (25 μM) in PBS (schwarz) und in wässrigen Suspensionen mit Liposomen (rot). *λ*_exc_: 530 nm (*n*=3). D) MIC‐Werte von Peptid **17** gegen verschiedene *Candida*‐Stämme und Bakterien (5×10^5^ Zelle mL^−1^) in 10 % flüssigen LB‐Medium nach 48 Stunden Inkubation bei 37 °C. Die MIC wurde durch Hellfeldmikroskopie aus drei unabhängigen Experimenten (*n*=3) bestimmt. e) Normalisierte Fluoreszenz der Probe **17** (30 μM) bei Inkubation in verdünnten Urinproben unter kontinuierlicher Lichteinstrahlung. *λ*_exc_: 530 nm. Daten dargestellt als Mittelwerte±SD (*n*=3).

Die Aktivität antimikrobieller Peptide als Schlüsselkomponenten des angeborenen Immunsystems macht sie zu attraktiven Vorlagen für translationale Anwendungen.^[30]^ Angesichts der kompakten Struktur und des fluorogenen Charakters der Phe‐BODIPY‐Aminosäuren untersuchten wir ihren Nutzen zur Herstellung fluorogener Peptide für den Nachweis von *Candida*‐Pilzzellen in klinischen Proben. Um mögliche Störungen durch die Autofluoreszenz biologischer Proben (z. B. Urin) zu vermeiden, haben wir die chemische Struktur von Phe‐BODIPY **6** feinabgestimmt, indem wir die beiden Methylsubstituenten durch verlängerte *p*‐Methoxyphenyl (*p*MP)‐Gruppen ersetzt haben, während alle anderen Positionen unberührt blieben (Abbildung 4a). Auf diese Weise haben wir die Phe‐BODIPY‐Aminosäure **10** über die Kupplung des TAM‐geschützten Alanin‐Surrogats **3** und des diaryliodierten BODIPY‐Derivats **8** durch eine Pd‐katalysierte C(sp^3^)‐H Arylierung, gefolgt von der Entschützung der *C‐* und *N‐*terminalen Schutzgruppen erreicht. Die resultierende Phe‐(*p*MP)BODIPY‐Aminosäure **10** behielt eine ausgezeichnete Fluorogenität mit maximalen Emissionswellenlängen um 620 nm (ergänzende Abbildung S10), was ein gutes Signal‐Rausch‐Verhältnis ohne Waschschritte begünstigt. Um die Integration von Phe‐(*p*MP)BODIPY als neuen Baustein für die Festphasen‐Peptidsynthese (SPPS) zu erleichtern, optimierten wir auch die Synthese eines Fmoc‐geschützten Aminosäure‐Surrogats (Verbindung **11**, Abbildung 4a). Es ist bekannt, dass antimikrobielle Peptide mit molekularen Komponenten der Zellmembran interagieren und sich in lipophilen intrazellulären Kompartimenten anreichern. Wie erwartet zeigte Verbindung **11** ein bemerkenswertes fluorogenes Verhalten, blieb in wässrigen Medien stumm (QY<0.001) und zeigte in lipophilen Mikroumgebungen eine um zwei Größenordnungen höhere Quantenausbeute (QY=0.15). Soweit wir wissen, ist Verbindung **11** das erste Beispiel für die Phe‐basierte BODIPY‐Aminosäure für SPPS mit starker Emission im roten Bereich des sichtbaren Spektrums. Vor dem Einbau der Phe‐(*p*MP)BODIPY‐Aminosäure **11** in Peptide, die *Candida*‐Pilzzellen aufspüren können, haben wir eine kleine Sammlung von Phe‐haltigen antimikrobiellen Peptiden als Pilz‐Zielgerüste vorbereitet, die spätere BODIPY‐basierte aromatische FIAAs aufnehmen könnten (Abbildung 4b). Wir wählten Peptidanaloga aus, die vom natürlich vorkommenden Temporin L (*Rana temporaria*, Peptid **12**),^[31]^ Jelleine‐I (*Apis mellifera*, Peptid **13**)^[32]^ und Aurein1.2 (*Litoria aurea* oder *Litoria raniformis*, Peptid **15**)^[33]^ abgeleitet sind. Diese kleine Datenbank wurde durch die Herstellung der unerforschten zyklischen Versionen der Peptide **13** und **15** vervollständigt, die Peptide **14** bzw. **16** ergeben. Die entsprechenden unmarkierten antimykotischen Peptide **12**‐**16** wurden mit Hilfe herkömmlicher Verfahren in SPPS^[34]^ synthetisiert und in ausgezeichneter Reinheit (>95 %) isoliert (vollständige Angaben zur Synthese und Charakterisierung in den Hintergrundinformationen). Wir untersuchten die Aktivität der Peptide **12**‐**16** in sechs verschiedenen *Candida*‐Stämmen (*C. krusei, C. albicans, C. glabrata, C. rugosa, C. auris* und *C. inconspicua*), die repräsentativ für Pilzinfektionen bei Krankenhauspatienten sind. Bei diesen Tests haben wir festgestellt, dass das Jelleine‐I Oktapeptid **13** bei den meisten *Candida*‐Stämmen die höchste biologische Aktivität zeigte, mit Arbeitskonzentrationen im niedrigen mikromolaren Bereich. Die Wirkungsweise von Jelleine‐I hängt von seiner Tendenz ab, in Lipiddoppelschichten von Pilzen in Form von *β*‐Faltblättern zu aggregieren.^[35]^ Interessanterweise führte die Zyklisierung des linearen Peptids **13** zum Peptid **14** zu einem vollständigen Verlust der Aktivität, was wahrscheinlich auf eine geringe Tendenz zur Bildung von β‐Strängen und zur Akkumulation in den Lipiddoppelschichten zurückzuführen ist. Daher synthetisierten wir das entsprechende Analogon des linearen Peptids **13** (Abbildung 4c, Peptid **17**), bei dem wir Phe aus der nativen Sequenz durch die Aminosäure Phe‐(*p*MP)BODIPY ersetzten (ergänzende Abbildung S11). Die Aminosäure **11** erwies sich als stabil gegenüber den Standardreaktionsbedingungen für die SPPS (z. B. Piperidin:DMF für die Fmoc‐Entschützung, DIC/COMU und OxymaPure für Aminosäurekupplungen), ohne dass ein Abbau der Phe‐(*p*MP)‐BODIPY‐Gerüst, die Kompatibilität der die Phe‐BODIPY‐Aminosäuren für die Synthese von fluoreszierenden Peptiden anzeigt. Nach dem Aufbau der Peptidsequenz führten wir die finale Abspaltung vom Sieber‐Amidharz unter mild‐sauren Bedingungen (<5 % TFA in DCM) durch, um die Integrität des BODIPY‐Kerns zu erhalten, und erhielten das gewünschte Peptid **17** nach der HPLC‐Aufreinigung (26 % Gesamtausbeute, 99 % Reinheit). Wie erwartet, zeigte das Peptid **17** ein gutes fluorogenes Verhalten mit starker Emission bei 620 nm (Abbildung 4c und ergänzende Abbildung S12). Als nächstes analysierten wir das biologische Profil von Peptid **17** in Pilzzellen und seine Selektivität gegenüber Bakterien. Die Bioaktivität von Peptid **17** ähnelte der des unmarkierten Peptids **13** mit niedriger mikromolarer Aktivität in verschiedenen *Candida*‐Stämmen (*C. inconspicua, C. albicans, C. krusei*) und minimaler Bioaktivität in Bakterienzellen (Abbildung 4d). Darüber hinaus untersuchten wir die photochemische Stabilität des Peptids **17** in menschlichem Urin bei kontinuierlicher Lichtbestrahlung. Bei diesem Test führten wir sowohl Fluoreszenz‐ als auch HPLC‐Analysen durch, und bestätigen, dass das Peptid **17** für die klinische Bewertung von Urinproben mit fluoreszenzbasierten Assays vollständig stabil war (Abbildung 4e und ergänzende Abbildung S13).

### Fluoreszenzbildgebung von Candida‐Zellen und Nachweis in Urinproben

**Figure 5:**
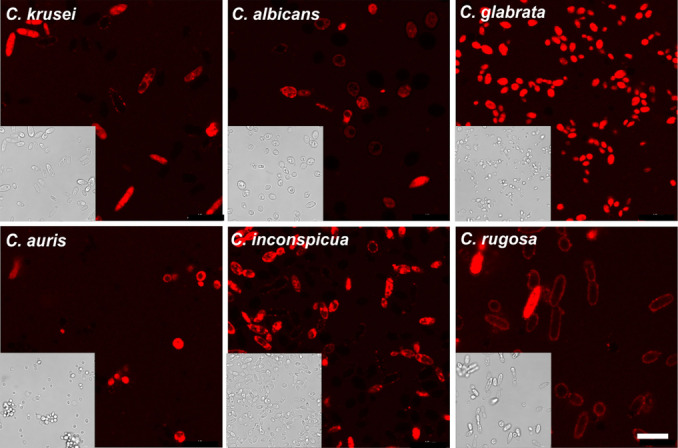
Konfokale Fluoreszenzmikroskopie von Lebendzellen verschiedener *Candida*‐Stämme nach Inkubation mit Peptid **17**. Hellfeld‐ (insets) und Fluoreszenzbilder (5×10^5^ Zellen mL^−1^) wurden nach einstündiger Inkubation mit Peptid **17** (10 μM) in PBS bei 37 °C ohne jegliche Waschvorgänge aufgenommen. *λ*_exc_: 575 nm, *λ*_em_: 600–650 nm. Maßstabsbalken: 10 μm.

Als nächstes führten wir konfokale Mikroskopieexperimente durch, um die potenzielle Anwendung des Peptids **17** zur Markierung lebender Kulturen von *Candida*‐Pilzzellen zu bewerten. Wir kultivierten Zellen der sechs verschiedenen *Candida*‐Stämme und bebrüteten sie mit der gleichen Konzentration des Peptids **17** (10 μM) unter physiologischen Bedingungen bei 37 °C. Hellfeld‐ und Fluoreszenzmikroskopische Aufnahmen wurden nach 1 h Inkubation ohne Waschschritte gemacht (Abbildung 5). Unter diesen Bedingungen konnten wir feststellen, dass alle *Candida*‐Stämme hell markiert waren und dass nur minimale Hintergrundsignale von Peptid **17** zu erkennen waren. Darüber hinaus beobachteten wir, dass Peptid **17** *C. glabrata*, C*. inconspicua* und *C. krusei*, hellere Signale zeigte, was mit den Ergebnissen aus den Bioaktivitätstests übereinstimmt (Abbildung 5 und ergänzende Abbildung S14). Wir bewerteten auch die Selektivität von Peptid **17** gegenüber *Candida*‐Zellen, indem wir seine Markierung mit anderen Bakterien‐ und Pilzstämmen verglichen, die üblicherweise in klinischen Proben vorkommen (*E.coli* bzw. *A. fumigatus*), die eine sehr schwache Markierung von Peptid **17** (ergänzende Abbildung S15).

**Figure 6:**
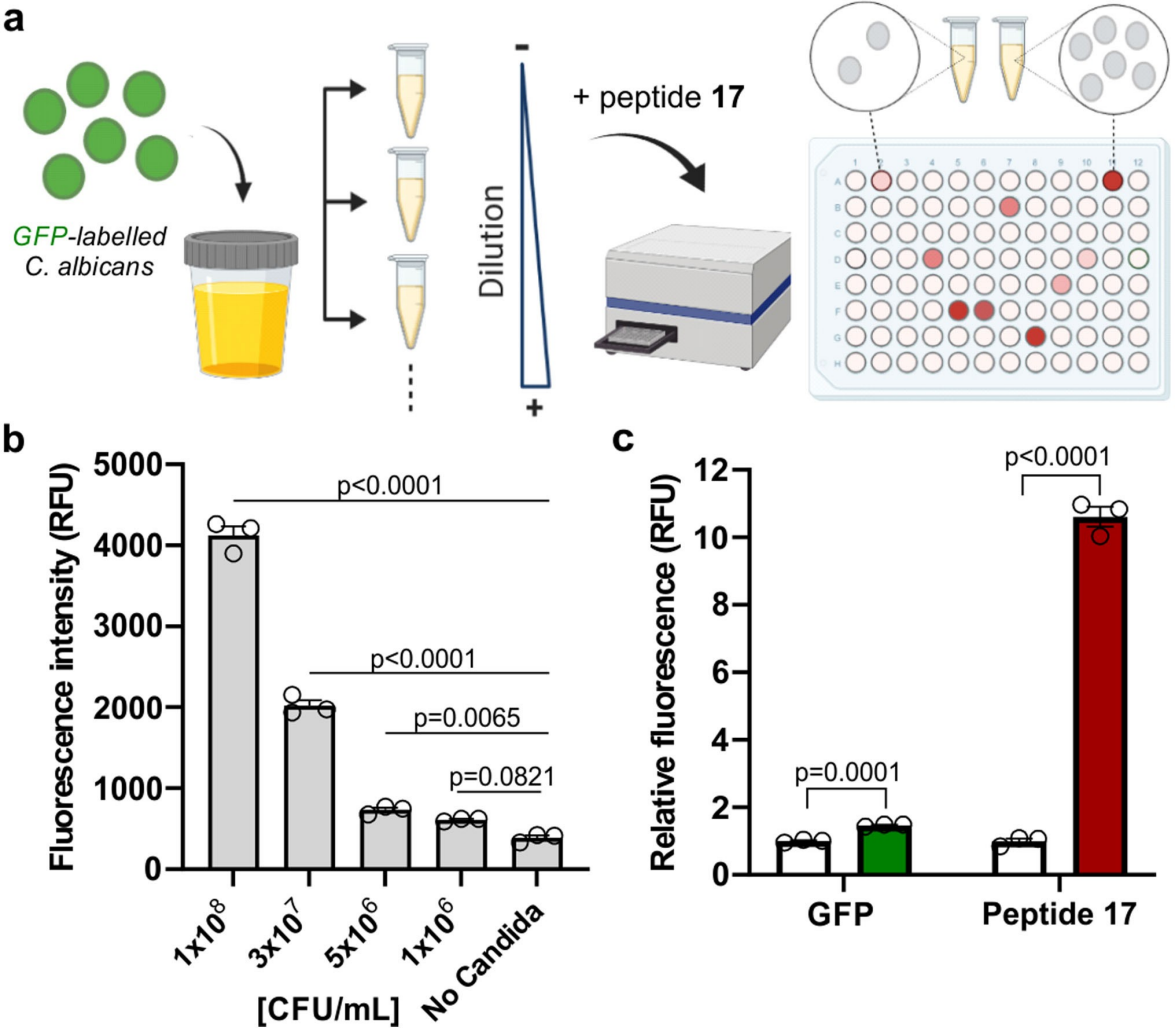
A) Schematische Darstellung der Quantifizierung des *Candida‐*Gehalts in Urinproben durch Fluoreszenzemission nach Inkubatioin Peptid **17** (10 μM) unter Verwendung eines Tisch‐Spektrophotometers. B) Fluoreszenzintensität von Peptid **17** (10 μM) nach Inkubation in Urinproben mit *C. albicans* im Bereich von 10^6^ bis 10^8^ CFU mL^−1^. Daten dargestellt als Mittelwert±SEM (*n*=3). P‐Werte aus ONE‐ANOVA‐Tests mit Mehrfachvergleichen. C) Relative Fluoreszenzintensität von GFP (grün) und Peptid **17** (rot) nach Inkubation in Urinproben mit *C. albicans* (10^8^ CFU mL^−1^) oder Urin allein (weiß). *λ*_exc_: 450 nm (GFP) und 530 nm (Peptid **17**), *λ*_em_: 515 nm (GFP) und 642 nm (Peptid **17**). Daten dargestellt als Mittelwert±SEM (*n*=3). P‐Werte wurden durch ungepaarte, zweiseitige t‐Tests ermittelt. Repräsentative Balkendiagramme aus zwei unabhängigen Experimenten mit drei technischen Replikaten.

Schließlich untersuchten wir, ob das rot‐emittierende fluorogene Peptid **17** zur quantitativen Messung von UT‐Pilzinfektionen in Urinproben eingesetzt werden kann. *C. albicans* ist der häufigste Stamm bei UT‐Pilzinfektionen,^[36]^ daher beschlossen wir, die Fluoreszenzwerte von menschlichem Urin zu messen, der mit grünem Fluoreszenzprotein (GFP) markierte *C. albicans*‐Zellen enthält, und sie mit denen zu vergleichen, die nach der Inkubation mit dem Peptid **17** erhalten wurden. Kurz gesagt wurden GFP‐exprimierende Pilzzellen in Urin in seriellen Konzentrationen von 10^6^ bis 10^8^ koloniebildenden Einheiten (KBE) pro mL verdünnt und dann in 384‐Well‐Platten plattiert, bevor sie 37 °C mit der Probe **17** inkubiert wurden (Abbildung 6). Nach einer Stunde Inkubation zeichneten wir die Fluoreszenzemission aller Vertiefungen bei 515 nm (GFP) und 642 nm (Peptid **17**) direkt in einem Tisch‐Spektrophotometer auf, ohne dass irgendwelche Verarbeitungs‐ oder Waschschritte erforderlich waren (Abbildung 6b und ergänzende Abbildung S17). Wir zeichneten Standardkalibrierungskurven für die beiden Fluoreszzenzanzeigen auf und bestimmten die entsprechenden Nachweisgrenzen (LoD). Nennenswert ist, dass das Peptid **17** eine bemerkenswerte Fluoreszenzantwort auf *C. albicans‐*Zellen mit einer LoD von 1.8×10^6^±0.6 CFU mL^−1^ zeigte, was eine Größenordnung niedriger ist als die mit dem GFP‐Readout (2.6×10^7^±0.4 CFU mL^−1^). Die Fluorogene Reaktion von Peptid **17** zeigte also eine mehr als 10‐fache Fluoreszenzzunahme im Gegensatz zum GFP‐Signal, das nur etwa 1,5‐fach war (Abbildung 6c). Schließlich synthetisierten wir auch die gleiche Peptidsequenz mit dem grün fluoreszierenden Trp‐BODIPY **4** (Peptid **18**, vollständige synthetische Details in den Hintergrundinformationen) als Negativkontrolle. Wie erwartet, zeigte Peptid **18** einen geringeren zweifachen Turn‐on‐effekt in *Candida*‐Suspensionen (ergänzende Abbildungen S18 und S19). Um die Kompatibilität von Peptid **17** mit Urinproben weiter zu bewerten, bestätigen wir auch das Fehlen einer potenziellen Kreuzreaktivität von Peptid **17** gegenüber reichlich vorhandenen Biomolekülen, die in Urin (z. B. Kreatinin, Harnstoff, Aminosäuren) (ergänzende Abbildung S20). Diese Ergebnisse bestätigen den Nutzen fluorogener antimikrobieller Peptide für den schnellen und kostengünstigen Nachweis von Pilzerregern in klinischen Proben und untermauern das Potenzial von **17** als Sonde für den Nachweis von *Candida*‐Zellen in menschlichen Urinproben.

## Zusammenfassung

Wir haben spektroskopische und computergestützte Methoden kombiniert, um die ersten fluorogenen Phe‐BODIPY‐Aminosäuren rational zu entwerfen und damit das derzeitige Instrumentarium an umweltempfindlichen fluoreszierenden Aminosäuren zu erweitern. Unsere Studie zeigt, dass sowohl das Substitutionsmuster als auch die Ladungstransfereigenschaften des BODIPY‐Kerns entscheidend für die Feinabstimmung des fluorogenen Charakters der Phe‐BODIPY‐FIAAs sind. Daher haben wir das Phe‐(*p*MP)BODIPY **11** als neuartigen rot‐emittierenden Baustein hergestellt, um fluoreszierende Peptide mit Hilfe konventioneller SPPS zu erzeugen. Nach der Identifizierung von antimykotischen Peptiden mit Affinität für *Candida*‐Zellen stellten wir das Peptid **17** als fluorogene Sonde für den schnellen und waschfreien Nachweis von *Candida*‐Pilzzellen in menschlichen Urinproben her. Peptid **17** weist eine gute Selektivität gegenüber bakteriellen Zellen und eine hohe chemische Stabilität auf und bietet eine einfache, empfindliche und kostengünstige Methode für den Nachweis von *Candiduria*. Die Vielseitigkeit, kompakte Größe und Fluorogenität von Phe‐BODIPYs wird die Herstellung anderer fluoreszierender Peptide ermöglichen, die den häufig vorkommenden Phe‐Rest tragen. Darüber hinaus haben wir Struktur‐Aktivitäts‐Beziehungen im Phe‐BODIPY‐Kern identifiziert, um die Energiebarrieren zu modulieren, die zu nicht‐strahlenden Zerfällen führen, was das zukünftige Design von BODIPY‐Bausteinen mit optimalen fluorogenen Eigenschaften beschleunigen wird.

## Interessenkonflikt

Die Autoren erklären, dass keine Interessenkonflikte vorliegen.

1

## Supporting information

As a service to our authors and readers, this journal provides supporting information supplied by the authors. Such materials are peer reviewed and may be re‐organized for online delivery, but are not copy‐edited or typeset. Technical support issues arising from supporting information (other than missing files) should be addressed to the authors.

Supporting Information

## Data Availability

Die Daten, die die Ergebnisse dieser Studie unterstützen, sind auf begründete Anfrage beim Autor erhältlich.
